# Optimizing Genomic Selection for a Sorghum Breeding Program in Haiti: A Simulation Study

**DOI:** 10.1534/g3.118.200932

**Published:** 2018-12-10

**Authors:** Kebede T. Muleta, Gael Pressoir, Geoffrey P. Morris

**Affiliations:** * Department of Agronomy, Kansas State University, Manhattan, Kansas; † Chibas and Faculty of Agriculture and Environmental Sciences, Quisqueya University, Port-au-Prince, Haiti

**Keywords:** Molecular breeding, genetic gain, genetic architecture, genome-wide prediction, simulation, smallholder agriculture

## Abstract

Young breeding programs in developing countries, like the Chibas sorghum breeding program in Haiti, face the challenge of increasing genetic gain with limited resources. Implementing genomic selection (GS) could increase genetic gain, but optimization of GS is needed to account for these programs’ unique challenges and advantages. Here, we used simulations to identify conditions under which genomic-assisted recurrent selection (GARS) would be more effective than phenotypic recurrent selection (PRS) in small new breeding programs. We compared genetic gain, cost per unit gain, genetic variance, and prediction accuracy of GARS (two or three cycles per year) *vs.* PRS (one cycle per year) assuming various breeding population sizes and trait genetic architectures. For oligogenic architecture, the maximum relative genetic gain advantage of GARS over PRS was 12–88%, which was observed only during the first few cycles. For the polygenic architecture, GARS provided maximum relative genetic gain advantage of 26–165%, and was always superior to PRS. Average prediction accuracy declines substantially after several cycles of selection, suggesting the prediction models should be updated regularly. Updating prediction models every year increased the genetic gain by up to 33–39% compared to no-update scenarios. For small populations and oligogenic traits, cost per unit gain was lower in PRS than GARS. However, with larger populations and polygenic traits cost per unit gain was up to 67% lower in GARS than PRS. Collectively, the simulations suggest that GARS could increase the genetic gain in small young breeding programs by accelerating the breeding cycles and enabling evaluation of larger populations.

High-yielding and climate-resilient crop varieties are needed to meet increasing demand for food and feed in the developing world (Tester and Langridge 2010; Crossa *et al.* 2014; Varshney *et al.* 2014; Bhat *et al.* 2016; Mausch *et al.* 2017). Young small breeding programs in developing countries face many constraints in genetic improvement of crop varieties, which include limited resources, multipurpose yield targets, and highly heterogeneous production environments. Due to slow breeding cycles, phenotypic selection approaches may not provide adequate genetic gain (Fischer *et al.* 2014; Bhat *et al.* 2016). This limitation is especially true for polygenic quantitative traits, which are controlled by large numbers of small effect loci and generally show low heritability (Varshney *et al.* 2014; Bhat *et al.* 2016).

Genomic selection (GS) has been evaluated in many crop improvement programs as an alternative to phenotypic selection (Habier *et al.*, 2009; Heffner *et al.*, 2009, 2010; Crossa *et al.*, 2010; Jannink *et al.*, 2010; Lorenz *et al.*, 2011; Hayes *et al.*, 2013; Lorenz 2013). In GS approaches, genome-wide prediction models are developed using markers that are in linkage disequilibrium (LD) with quantitative trait loci (QTL); the models of marker effects are used to calculate genomic-estimated breeding values (GEBVs) for genotyped lines and the GEBVs are used for selection (Meuwissen *et al.* 2001). Once trained with field phenotypes, GS allows selection without field evaluation, so genetic gain can be increased through increased population size and selection intensity. GS can accelerate population improvement and lower the cost of identifying genotypes with improved breeding or varietal value (Bernardo and Yu 2007; Crossa *et al.* 2014, 2017; Bassi *et al.* 2016; Bhat *et al.* 2016; Zhang *et al.* 2017).

GS is generally applied to facilitate recurrent selection (RS) schemes (Bernardo and Yu 2007; Grenier *et al.* 2015; Hickey *et al.* 2017; Müller *et al.* 2017; Morais Júnior *et al.* 2018). RS is a cyclical breeding approach for improvement of quantitative traits in breeding populations. RS produces novel gene combinations and increases the frequency of favorable alleles (Windhausen *et al.*, 2012; Müller *et al.*, 2017). Phenotypic recurrent selection (PRS) involves three steps conducted in a cyclical manner: (i) development of progeny (S0) by crossing selected parents, (ii) evaluation of progeny (S1 or S2) to select new parents, and (iii) recombination of superior progeny to create the next cycle (Weyhrich *et al.* 1998). Accurate evaluation of progeny performance is limited by lack of seed for replicated trials. Genomic-assisted recurrent selection (GARS) would allow prediction of S0 breeding values without evaluation of S1 or S2 families in replicated trials.

There is great interest and potential to apply GS in developing country breeding programs (Varshney *et al.* 2014; Bhat *et al.* 2016; Crossa *et al.* 2017; Xu *et al.* 2017). However, most studies intended to guide GS implementation were not parameterized to reflect small developing country breeding programs (Bernardo and Yu 2007; Lorenz 2013; Müller *et al.* 2017). GS implementation should take into account these programs’ unique challenges/advantages and selection histories. For instance, the Fisher-Orr model of adaptive walks would predict that young breeding programs have more large effect variants segregating, while established breeding programs (which have fixed large effect variants) should approach an infinitesimal model (Orr 2009). Simulation studies can been used to investigate genetic gain, prediction accuracy, cost-effectiveness of GS under various scenarios (Bernardo and Yu 2007; Habier *et al.* 2007; Heffner *et al.* 2009; Solberg *et al.* 2009; Jannink 2010; Bastiaansen *et al.* 2012; Müller *et al.* 2017). However, to our knowledge, no simulation study has evaluated GS *vs.* phenotypic selection considering breeding population size, genetic architecture and costs of small breeding programs in developing countries.

In most developing countries of the semi-arid tropics, including Haiti and countries in sub-Saharan Africa, sorghum is grown primarily under low-input rain-fed conditions with heterogeneous climate and soil conditions (Kumar *et al.*, 2011). Here, our goal was to evaluate GS strategies for the Chibas sorghum breeding program in Haiti and similar breeding programs. The Chibas sorghum program develops pure line varieties for low-input smallholder agriculture in Haiti using RS with a *ms3* genetic male sterility (Brocke *et al.* 2008). The program was launched in 2011 with two rounds of intermating of founders (selections from global tropical germplasm) to establish a base population (Cycle 0). PRS based on evaluation of S1 and S2 families began in 2013. The PRS program proceeded to cycle 3 by 2016 when population improvement was paused to develop sugarcane aphid (*Melanaphis sacchari*) tolerant varieties. A shift to GARS would allow the prediction of breeding value on S0 progenies, potentially shortening the breeding cycle to one generation. Further, GARS could take advantage of the tropical environment to rapidly cycle two to three times per year. The objective of this simulation study was to compare genetic gain, prediction accuracy, genetic variance, and cost under GARS *vs.* PRS across various genetic architectures, breeding population sizes, and model updating scenarios. We found that by accelerating the breeding cycle and enabling evaluation of larger populations GARS could increase genetic gain and lower cost per unit gain in developing country breeding programs.

## MATERIALS AND METHODS

### Overview of breeding program simulation

**Figure 1:**
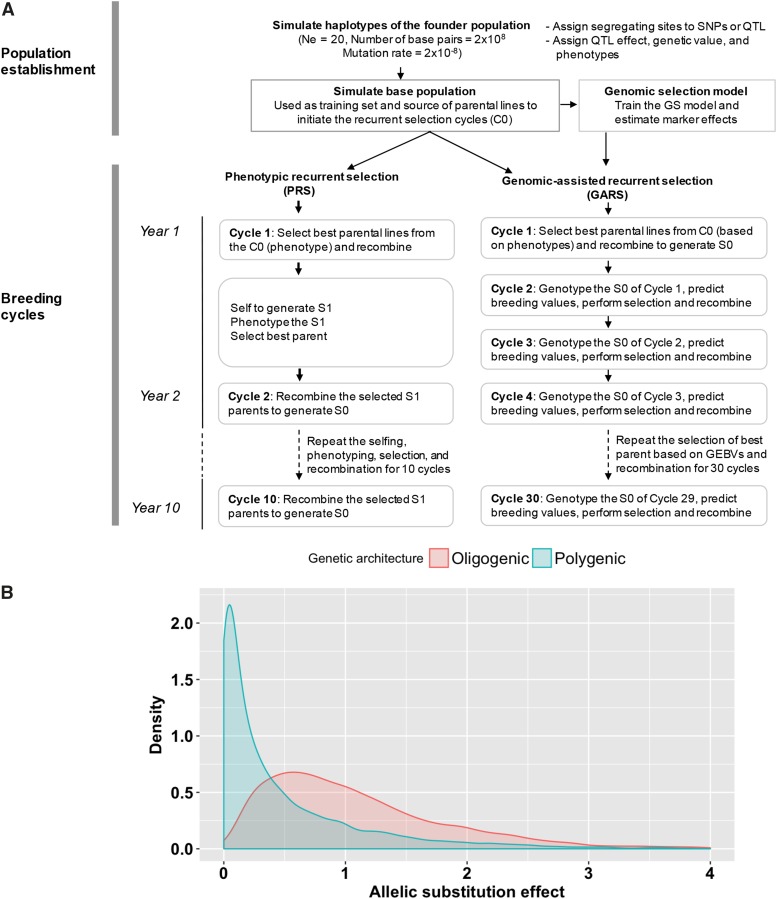
Breeding program simulation. (A) Schematic representation of the simulated breeding programs. In the population establishment phase, the base population was created by randomly intercrossing the founder population for several generations, which was then phenotyped and genotyped to be used as a training population. For the breeding cycles, the training population was used as a cycle 0 generation from where best parents were selected to initiate PRS and GARS. GARS was conducted three cycles per year (GARS3, shown) or two cycles per year (GARS2, not shown), compared to a single cycle of PRS per year. (B) The density plot of the distribution of allelic substitution effects of the quantitative trait loci (QTL) simulated in two trait genetic architectures (oligogenic and polygenic architectures).

The Chibas sorghum breeding program’s RS scheme is based on the evaluation of S1 progenies, which involves the selection of best parents followed by randomly intermating the selected parents to generate the next selection cycle. Each cycles of RS followed by multiple generations of selfing to generate inbred line. The inbred families will be evaluated in replicated trials in multiple locations toward variety release. The Chibas sorghum breeding program was simulated using AlphaSimR package (https://cran.r-project.org/web/packages/AlphaSimR/index.html) (Faux *et al.* 2016) (Figure 1). All results are based on 100 simulated breeding programs, each from an independently simulated founder population. Haplotype sequences of founder populations were simulated with the coalescent simulation program MaCS (Chen *et al.*, 2009) implemented in AlphaSimR. The population parameters used to simulate the founders include effective population size (Ne) of 20 (the approximate number of founders of the Chibas program), number of base pairs = 2x10^8^, and mutation rate = 2x10^−8^ (Figure 1A). Genomes were defined with 10 chromosomes, as in sorghum (Paterson *et al.* 2009), and 1,000 segregating sites per chromosome. Segregating sites in the founder population were assigned randomly to become either markers (n = 9,000) or QTL (n = 1,000). The actual number of QTL with effect size greater than zero varied according the genetic architecture of the trait simulated.

### Defining trait genetic architectures

Traits were simulated with (i) oligogenic or (ii) polygenic architecture by varying the number and effect size of QTL (Figure 1B). The oligogenic and polygenic traits were assumed to have high and low heritability, respectively, to represent contrasting extremes of genetic architecture. Oligogenic architecture was simulated by randomly sampling 50 QTL (five per chromosome) with effects sizes drawn from a gamma distribution with scale and shape parameter of 0.5 and 1.8, respectively. Components of phenotypic variance (additive genetic variance, environmental variance, genotype by environment interaction [GxE] variance, and residual variance) were chosen to make heritability *h*^2^ = 0.7. For the oligogenic trait, we are referring to a quantitative trait that is controlled by relatively small number of genes, in which most of the trait variation is attributed to the top few genes. In this case, only five loci are responsible for 45% of the trait variation.

Polygenic architecture was simulated by sampling 500 QTL (50 per chromosome) with effect sizes drawn from a gamma distribution with scale and shape parameter of 1.0 and 0.5, respectively. Variance components were chosen to make heritability *h*^2^ = 0.3. Intra- and inter-locus interactions (dominance and epistasis effects) were not modeled. For each of the two genetic architectures, various population sizes and selection schemes were compared for genetic gain, genetic variance, and prediction accuracies. True breeding value for each individual was calculated by summing effects across all QTL. Genetic gain (assuming true breeding value of zero at *t* = 0) was defined and plotted as the true breeding value expressed in standard deviation units of the phenotypic values of cycle 0 (C0) generation.

### Defining base/training populations

For each simulation, a base population (C0 generation) of 400 inbred lines was simulated by randomly mating the founder population, followed by five generations of selfing to create inbred lines. Phenotypic values for each inbred line in the base population was simulated by adding normally-distributed random deviates to each line’s true breeding value. The base population was used as a source of parental lines that were used to create the initial recurrent selection population. The base population is also used as the training population for estimation of markers effects for the genome-wide prediction model.

### Conducting phenotypic recurrent selection

PRS was performed by selecting the top 5, 20, 100, or 200 parental lines from the base population based on their phenotypic values. Selected parents were randomly intermated to generate S0 individuals. The S0 individuals were then selfed to generate S1 families. Following the Chibas breeding program’s current PRS method, the S1 families were phenotyped and S1 families with the top phenotypic values were randomly intermated to generate the next cycle. Intermating of 5, 20, 100, or 200 parents generated 50, 200, 1000, or 2000 S0 individuals, respectively (designated par_5__pop_50_, par_20__pop_200_, par_100__pop_1000_ and par_200__pop_2000_, respectively) (File S1A). In all of these scenarios, the top 10% of S1 families were retained each cycle. Since developing countries breeding programs generally have limited field phenotyping capacity, scenarios were also simulated where the number of phenotyped S1 families was capped at 500. In these scenarios (designated as par_100__pop_500_, par_200__pop_500_), populations were generated by randomly intermating 100 or 200 selected parents to generate 500 S0 individuals, which meant the top 20% or 40% of S1 families were retained, respectively. Assuming that the first selfing generation (S0→S1) was performed during an off-season nursery, a single cycle of PRS was performed each year, for a total of ten cycles of PRS in ten years. For each cycle of PRS, genetic gain, genetic variance, and prediction accuracy were calculated. Prediction accuracy in PRS was calculated as the Pearson correlation between the phenotypic value and true breeding value of the S1 families.

### Conducting genomic-assisted recurrent selection

Similarly, GARS was initiated by selecting the top 5, 20, 100, or 200 parental lines from the base population based on their phenotypic values and randomly intermating to generate 50, 200, 1000, or 2000 S0 populations, respectively. Ridge regression best linear unbiased prediction (RR-BLUP) (Endelman 2011) was used to estimate genome-wide marker effects (using the phenotypic and genotypic data from the training population) and calculate GEBVs of the S0 individuals according to the following model:

$$y_{i}=\mu +(\overset{p}{\underset{k=1}{\sum }}X_{ik}\beta_{k})+e_{i}$$

where *y_i_* is the phenotype for individual *i*; *μ* is the overall mean; *X_ik_* is the genotype of individual *i* at marker *k*; *β_k_* is the additive effect of marker *k*; *p* is the number of markers; and *e_i_* is the residual effect. GEBVs were estimated using the following formula: *y_v_* = **M***β*, where *y_v_* is an *n*×1 vector of GEBVs for the accessions in the training population, **M** is an *n*×*m* matrix of genotype indicators for the training population, *β* is an *m*×1 vector of marker effects, *n* is the number of individuals, and *m* is the number of markers.

The GEBVs were used to select the top S0 individuals of C1 population. The selected S0 individuals were randomly intermated to generate the next cycle (C2) recurrent selection population, and so on for 30 cycles. To assess the effect of number of breeding cycle per year, two or three cycles of GARS per year (designated GARS2 and GARS3, respectively) were compared to PRS. Note, the results for GARS2 and GARS3 represent the same simulation runs, plotted differently to represent the given number of GARS cycles per year. Therefore, given a 10-year time horizon, there were 20 cycles of GARS2 and 30 cycles of GARS3. To align with the 10-year selection cycles of the PRS, only the first 28 and 19 cycles were plotted. During each cycle, the number of parents and populations generated by random intermating of the parents remained constant. Genetic gain, genetic variance, and prediction accuracy were calculated for the different selection schemes, population sizes, and trait genetic architectures. Prediction accuracies in GARS was calculated using a Pearson correlation between the GEBVs and true breeding values of S1 families.

### Updating training populations and prediction models

We examined the impact of updating the training populations and prediction models on the genetic gain, genetic variance, and prediction accuracy. Given that cropping-season phenotyping can only be conducted once per year, updating of the training population and prediction model was performed every two and three cycles for GARS2 and GARS3, respectively. The updated training population for the a given cycle (*N*_ind_ = 400) consisted of (i) the 200 top lines from the previous training population based on phenotype values and (ii) the 200 top lines from the previous cycle based on GEBVs. Updating of the training population was performed based on the population size of par_100__pop_1000_, which represents an optimum breeding population size at which genetic gain has plateaued. Changes in genetic gain, genetic variance, and prediction accuracy due to updating were compared to no-update GARS scenarios and PRS.

### Comparing costs of GARS *vs.* PRS

We compared the efficiency of the two selection methods in terms of cost per unit gain. One cycle of PRS in the Chibas sorghum breeding program involves the intercrossing of best parents to generate S0 individuals; planting and selfing of the S0 individuals to generate S1 families; and evaluation of the S1 families in replicated trials. The following costs (in US dollars per line calculated from 2017/18 budget estimates from Chibas sorghum breeding program) were assumed: $38 in the crossing phase, $14 in managing the S0 phase, and $33 replication^-1^ for evaluating the S1 families (File S1B). Costs in PRS were calculated for the following activities; planting and field management, pollination, perform replicated yield trial for grain yield, disease resistance/tolerance, stay-green, stem biomass, stem sugar content and other traits. GARS does not require phenotypic evaluation of the S1 population, but estimates breeding values of the S0 individuals. The costs involved in the different activities GARS includes genotyping and phenotyping of the training population, crossing of the parents and genotyping of the S0 individuals. Different genotyping cost, which include US $5, $15, $25, and $35 were used to estimate cost per gain for GARS and compared to that of PRS. Cost per unit genetic gain for the different scenarios were calculated by dividing the cumulative cost per cycle with the corresponding genetic gain.

### Data availability

All relevant data are within the paper and its supplemental files. Figure S1 shows the effects of updating the training population under an polygenic architecture. Figure S2 and S3 describe cumulative cost per unit gain in GARS2 and GARS3, respectively, compared to PRS. Figure S4 shows the costs per unit gain for updating the training population. Figure S5 shows the shift in the trait genetic architecture due to selection. File S1 summarizes the different populations sizes and cost estimates of activities in GARS and PRS. File S2 presents the *t*-tests for comparing the genetic gain between GARS and PRS across the different trait genetic architectures and population size. File S3 contains the script used to simulate the breeding program, while File S4 contains the script used to run the cost analysis and the output from the breeding program simulation (genetic gains) that was used to run the cost analysis. Supplemental material available at Figshare: https://doi.org/10.25387/g3.7396781.

## RESULTS

### Genetic gain differences among scenarios

**Figure 2:**
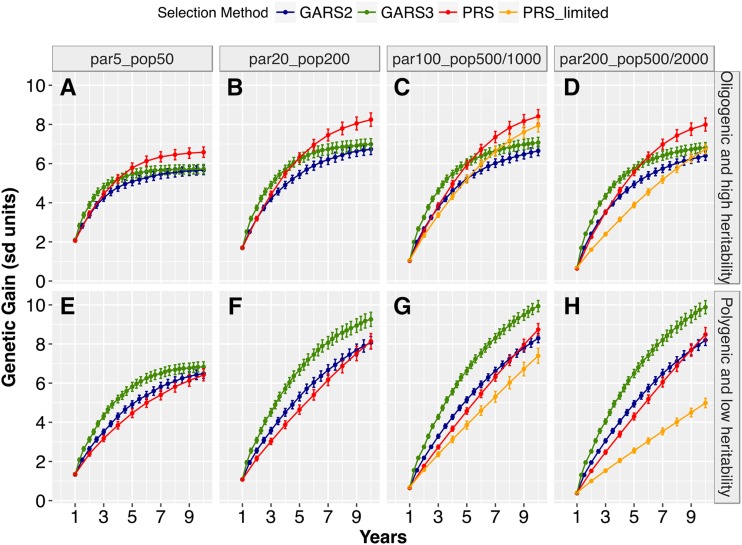
Genetic gains from genomic assisted recurrent selection (GARS) *vs.* phenotypic recurrent selection (PRS). The two selection schemes were compared across five population sizes and two types of traits genetic architecture (oligogenic and high heritability *vs.* polygenic and low heritability). The five population sizes were designated as par_5__pop_50_, par_20__pop_200_, par_100__pop_1000_, par_200__pop_2000_, which represent selection of 5, 20, 100, or 200 best parents and randomly intermated to create 50, 200, 1000, or 2000 individuals, respectively. par_100__pop_500_ and par_200__pop_500_ represent the selection of 100 and 200 best parents and randomly intermated to create 500 individuals, respectively. Changes in genetic gain (Y-axis) was ploted against selection cycles in years (X-axis). The plotted values are means (+/− 95% confidence interval) from 100 simulations.

We first compared the genetic gain across population sizes, genetic architectures, and selection schemes. The genetic gain per unit time resulting from GARS and PRS differed significantly and depended on population size and genetic architecture (Figure 2; *t*-tests in File S2). For the oligogenic architecture, GARS3 yielded significantly higher (*P* < 0.01) short-term genetic gain (year < 5) than PRS for all population size (Figure 2A-D). Across the years, the maximum relative short-term genetic gain from GARS3 compared to PRS ranged from 12–88% over different population sizes. By contrast, GARS2 yielded short-term genetic gain comparable to that of PRS. Long-term genetic gain (year ≥ 5–6) for the oligogenic architecture was significantly higher (*P* < 0.01) for PRS compared to either GARS2 or GARS3. For the polygenic architecture, GARS3 resulted in higher genetic gain (*P* < 0.01) over PRS for all population sizes across all selection cycles, except the first year (Figure 2E-H). Across years, maximum relative advantage of GARS3 over PRS was 26–165% depending on population size. Similarly, GARS2 provided higher genetic gain (*P* < 0.01) than PRS across all population sizes for the polygenic architecture, with maximum relative advantage of 12–99%.

For both genetic architectures, increasing population size resulted in increased genetic gain with GARS and PRS. Given GARS3 and oligogenic architecture, final genetic gain (year 10) increased by 22% and 3% when population size was increased (from par_5__pop_50_ to par_20__pop_200_ and from par_20__pop_200_ to par_100__pop_1000_, respectively; Figure 2A-C). Given PRS and oligogenic architecture, final genetic gain increased by 25% and 2%, respectively, for the same population size increases. Further increase in population size (to par_200__pop_2000_) did not result in significant increase in genetic gain (Figure 2C *vs.* 2D). For polygenic architecture, final genetic gain from GARS3 increased by 36% and 7%, for the same population size increases. (Figure 2E-G). With the exception of the smallest population size, GARS3 genetic gain (under polygenic architecture) continued to increase through the final year.

### Genetic variance differences among scenarios

**Figure 3:**
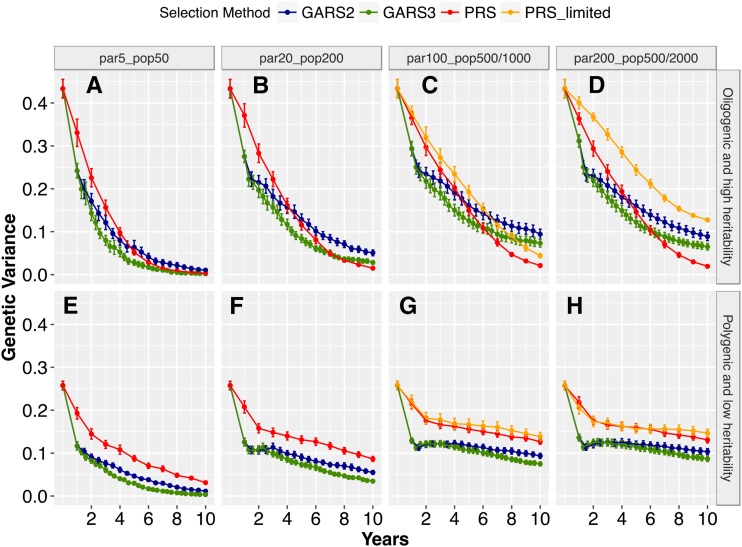
Changes in genetic variance during the recurrent selection process. Genomic assisted recurrent selection (GARS) was compared to the phenotypic recurrent selection (PRS) across five population sizes and two types of traits genetic architecture (oligogenic and high heritability *vs.* polygenic and low heritability). The five population sizes were designated as par_5__pop_50_, par_20__pop_200_, par_100__pop_1000_, par_200__pop_2000_, which represent selection of 5, 20, 100, or 200 best parents and randomly intermated to create 50, 200, 1000, or 2000 individuals, respectively. par_100__pop_500_ and par_200__pop_500_ represent the selection of 100 and 200 best parents and randomly intermated to create 500 individuals, respectively. Changes in total genetic variance (Y-axis) was ploted against selection years (X-axis). GARS2 and GARS3, with two and three cycles of genomic selection per year, respectively, were compared to one cycle PRS per year. Plotted values are means (+/− 95% confidence interval) from 100 simulations.

Total genetic variance was reduced over generations with both GARS and PRS (Figure 3). The decline in genetic variance differed highly significantly based on population sizes and genetic architecture (*P* < 0.001). For the oligogenic architecture, genetic variance during early selection years (year < 5) were higher for PRS compared to GARS (Figure 3A-D). In the later years (year ≥ 5), differences in genetic variance between the selection schemes became (i) non-significant (*P* > 0.05) at the smallest population size or (ii) significantly higher (*P* < 0.01) for GARS than PRS at the largest population size, notably for GARS2. Under the assumptions of limited phenotyping capacity in PRS (maximum population size capped at 500), genetic variance was higher than GARS at the maximum population size (*P* < 0.01; Figure 3D). Genetic variance declined to zero in PRS for all population sizes (Figure 3A-D) and at the smallest population size with GARS2 and GARS3 (Figure 3A).

The decline in genetic variance over years was minimal for the polygenic architecture, particularly for the larger population sizes (Figure 3E-H). Genetic variance was higher for PRS than GARS2 and GARS3 across all population sizes and selection years (*P* < 0.01). This difference was greater during the early years, but declined in later years. Similarly, the difference in genetic variance between GARS2 and GARS3 was minimal during the early years, but GARS3 lost more variance than GARS2 in later years (*P* < 0.01). For the polygenic architecture, genetic variance declined to zero only for GARS3 at the smallest population size (Figure 3E).

### Prediction accuracies differences among scenarios

**Figure 4:**
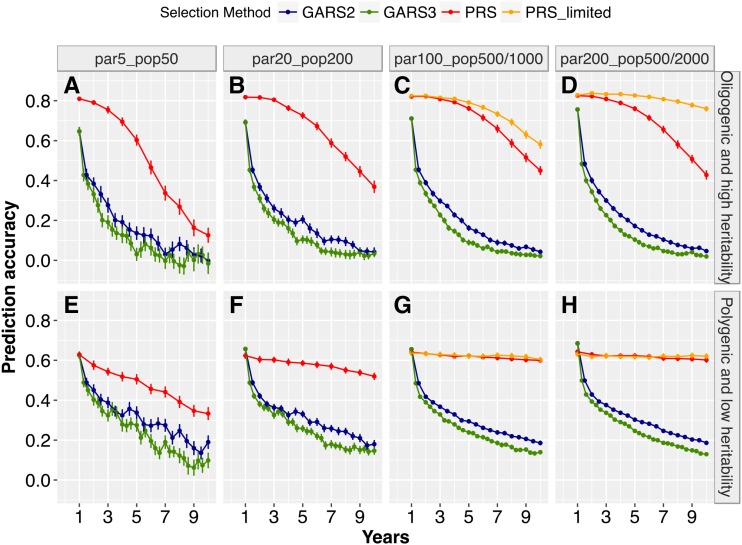
Effects of genetic architecture on prediction accuracy. Average prediction accuracy of genomic estimated breeding values (GEBV) under genomic-assisted recurrent selection and phenotypic estimated values (PEV) under phenotypic recurrent selection across years of selection for different breeding population sizes and trait genetic architectures, for an oligogenic and high heritability trait or polygenic and low heritability trait. The Y-axis represent accuracy of predicting GEBVs (estimated as the correlation between the GEBVs and true breeding values) and PEV (estimated as a correlation between the phenotypic-estimated values and true breeding values), while the X-axis represent selection years. The plotted values are means (+/− 95% confidence interval) from 100 simulations.

Prediction accuracy of GARS declined over generations, at rates that varied by trait genetic architecture and population size (Figure 4). In early years, average GARS prediction accuracy was higher for the oligogenic architecture (0.64–0.76) compared to that of polygenic architecture (0.62–0.68) (Figure 4A-D). However, the decline in prediction accuracy given oligogenic architecture was more rapid than that given polygenic architecture, notably after the second year of RS. Accordingly, prediction accuracies were significantly higher (*P* < 0.01) given polygenic architecture *vs.* oligogenic architecture in the later years (Figure 4B-D). PRS prediction accuracy for the oligogenic architecture was initially high but declined over years, most notably for the smallest population size (Figure 4A-D). For the polygenic architecture, PRS prediction accuracy showed strong decline only for the smallest population size (Figure 4E-H).

### Impact of updating the training population and prediction model

**Figure 5:**
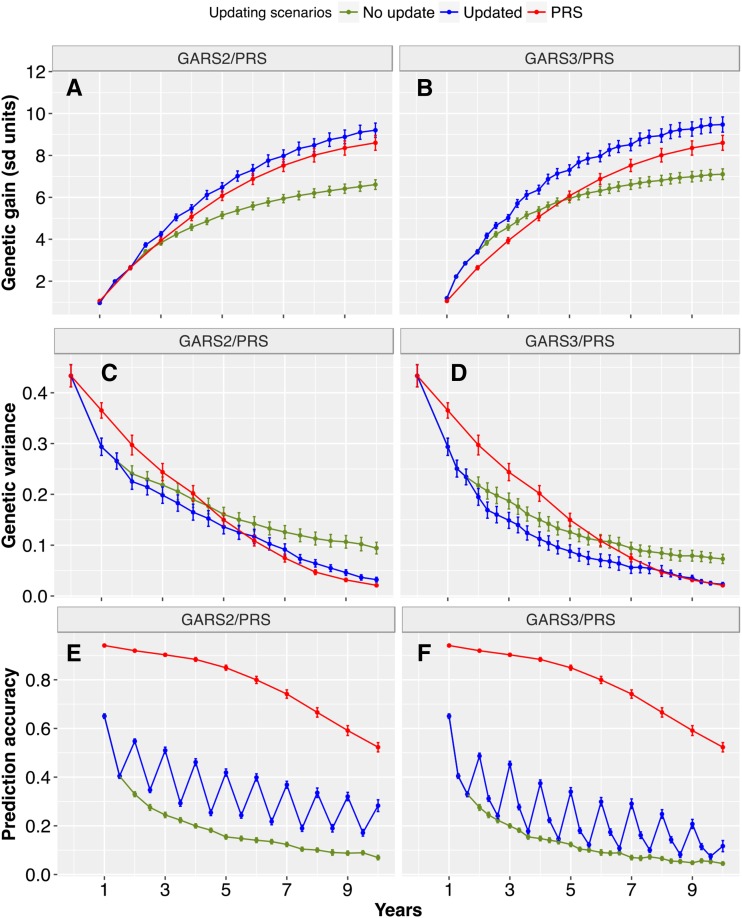
Effects of updating the training population on genetic gain, genetic variance and prediction accuracy. Genetic gain (panels A and B), genetic variance (panels C and D), and prediction accuracy (panels E and F) for GARS on oligogenic and high heritability trait when the training population was updated and the model retrained every two cycle (GARS2) (left panels) or three cycles (GARS3) (right panels) and compared to no update scenario. Updating was performed by mixing 200 best performing individuals from the recurrent selection cycle with 200 best performing individuals from the training population. The recurrent selection was performed by selecting 100 best performing individuals and intermated to create 1000 recurrent selection population, which was carried out for 30 cycles.

Given the decline of prediction accuracy with GARS, we considered the effects of updating the training population and prediction models to account for new LD generated over breeding cycles. We simulated scenarios with updates each year (every two or three cycles for GARS2 and GARS3, respectively) and compared these scenarios to no-update GARS and PRS (Figure 5 and Figure S1). Updating strategies provided large increases in genetic gain in later years of GARS under both oligogenic (Figure 5) and polygenic architectures (Figure S1). For the oligogenic architecture, final genetic gain increased by 39% and 7% for updated GARS2, compared to no-update GARS2 and PRS, respectively (Figure 5A). For updated GARS3, genetic gain increased by 33% and 10% over no-update GARS3 and PRS, respectively (Figure 5B).

Given polygenic architecture, final genetic gain (year 10) was increased by 29% and 13% for updated GARS2 compared to no-update GARS2 and PRS, respectively (Figure S1A). For updated GARS3, the increase in final genetic gain was 30% and 40% compared to no-update GARS3 and PRS, respectively (Figure S1B). Updating resulted in a greater decline of genetic variance compared to no-update GARS (Figure 5C-D). Prediction accuracy remained largely above 0.2 with updated GARS2 and GARS3 (Figure 5E-F).

### Cost per unit gain differences among scenarios

**Figure 6:**
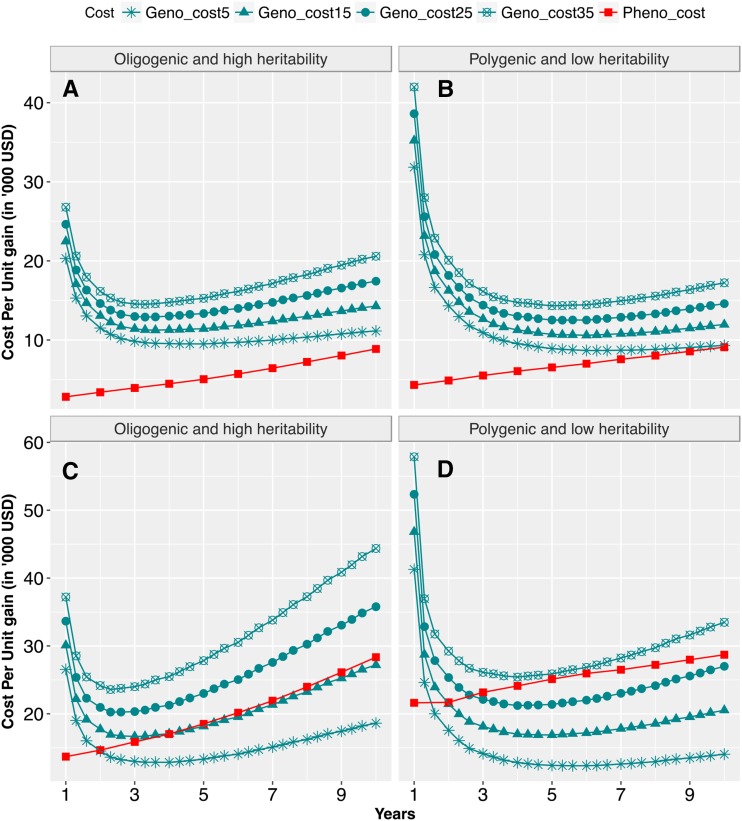
Cumulative cost (in thousands of US dollars) per unit gain in GARS3 compared to PRS: Four different genotyping costs (turquoise color) were compared to that of PRS (red color) for oligogenic architecture and polygenic architecture for population sizes of par_5__pop_50_ (panels A and B), and par_20__pop_200_ (panels C and D). The four realistic genotyping costs include $5, $15, $25, and $35 per sample; were compared to phenotyping costs for PRS, which was calculated based the current Chibas expenses for managing field experiments.

Cost per unit genetic gain was compared for GARS *vs.* PRS across population sizes, genetic architectures, and updating scenarios. For small population size, cost per unit gain was lower than GARS regardless of trait genetic architecture and genotyping costs considered. Cost per unit gain was lower by up to 67% in GARS than PRS when larger population size was evaluated, notably at the genotyping cost of $15 or under (Figure 6; Figure S2; Figure S3). When updating the training population, the additional cost of evaluating the training population was offset by the extra genetic gain achieved by model retraining (Figure S4B, C, D, G, and H). The exception was at the early selection years of updating GARS2 in which cost per unit gain in no-update GARS2 was slightly lower than that of updated GARS2. Given genotyping cost of $15 or lower, cost per unit gain in updated GARS2 was lower than PRS Hence, cost per unit gain in updated GARS2 and GARS3 was either comparable to or lower than no-update scenarios, except at early years of the selection cycles.

## DISCUSSION

### Maximization of genetic gain

Increasing genetic gain is a top consideration for breeding program design in the Chibas sorghum program and many similar programs (Xu *et al.* 2017). Simulation and empirical studies have reported that GS increases genetic gain, but have not been specifically parameterized to reflect small breeding programs in developing countries (Bernardo and Yu 2007; Lorenzana and Bernardo 2009; Heffner *et al.* 2010). Our simulation of genetic gain in GARS *vs.* PRS was designed to represent the genetic architecture and breeding schemes of the Chibas sorghum program in Haiti, but may be relevant to programs with similar genetic properties. The simulations show that GARS should, in most cases, increase genetic gain compared to PRS when at least two cycles of GARS are performed. Given oligogenic architecture and no updates of GARS models, however, the long-term genetic gain from PRS is expected to be greater than GARS. This result is likely due to new variation released by recombination that can be selected with PRS but not with no-update GARS. When the GARS prediction model is updated regularly, GARS outperforms PRS under either genetic architecture. Together, these findings highlight the likely advantage of GARS and the value of GARS model updating.

Larger population sizes provide the opportunity to increase selection intensity while maintaining genetic variance (Guzman 1998; Jannink 2010; Lorenz 2013; Gorjanc *et al.* 2017; Xu *et al.* 2017). In our simulation, genetic gain was significantly greater in larger breeding populations (*P* < 0.01, as described in previous simulations (Lorenz 2013; Slater *et al.* 2016; Xu *et al.* 2017). In practice however, population size is limited by capacity to manage field experiments. When a realistic limit on phenotyping capacity for Chibas was imposed, the advantage of GARS over PRS increased by more than fourfold. The effect of increased populations size may also be dictated by the genetic architecture of the trait. Increasing population size increased long-term gain given polygenic architecture, but provided little benefit given oligogenic architecture.

The advantage of GARS over PRS may vary according to the selection history of the breeding program and concomitant changes in genetic architecture (Roff and Mousseau 1999; Kumar *et al.* 2008). Breeding programs that have had few selection cycles (as in the case of Chibas sorghum breeding program) are expected to segregate large effect variants, while breeding programs that have had many selection cycles should segregate small effect loci (Orr 2009). Given oligogenic architecture in a young breeding program, GARS may provide only a short-term increase in genetic gain. However, as large-effect variants are fixed genetic architecture may become more polygenic (Figure S5), so GARS may provide both short- and long-term increase in genetic gains. That is, benefits of implementing GARS may increase as the selection cycles advance the program toward the infinitesimal model.

### Maximization of cost effectiveness

Cost-efficiency of evaluating and maintaining the breeding population is also a critical aspect of implementing genomic-assisted recurrent selection (Bernardo and Yu 2007; Wong and Bernardo 2008; Varshney *et al.* 2014; Bassi *et al.* 2016; Bhat *et al.* 2016; Crossa *et al.* 2017). We compared cost per unit gain in GARS *vs.* PRS across various population size and trait genetic architecture. The relative cost effectiveness of GARS *vs.* PRS was sensitive to many aspects of the breeding scheme. For instance, GARS was more cost efficient than PRS given current cost of field phenotyping for Chibas, moderate breeding population size (par_20__pop_200_ or larger), and genotyping cost <$15 per sample. With higher genotyping costs, the greater cost efficiency of GARS will be due to larger breeding population (par_100__pop_1000_ and par_200__pop_2000_). The result implied that it would be economically feasible to implement GARS to Chibas breeding program even with relatively expensive genotyping costs (US $15/sample) and moderate breeding population size. When genotyping costs are driven down to $5/sample, GARS is expected to be cost competitive even when traits are oligogenic and highly heritable.

Despite recent genotyping advances, existing platforms may not be appropriate for GARS. For instance, genotyping-by-sequencing (GBS) has been used to rapidly generate 100,000-500,000 SNP markers for diverse sorghum germplasm (Bekele *et al.* 2013), but the cost (∼$20–40 USD), turn-around time (∼6–8 weeks), and bioinformatics requirements may be prohibitive for GARS. One option would be to use GBS and resequencing data to develop an out-sourced SNP assay with 2,000–10,000 informative markers. If a turn-around of <4 weeks and cost of $5–10/sample was achieved, our simulations suggest that GARS would be effective in developing country breeding programs. Given the volume of data production and turn-around times, it would be critical to provide user-friendly bioinformatics and decision-support tools as a part of a breeding management system (Delannay *et al.* 2012; Varshney *et al.* 2015). Finally, training of developing country breeders in genetic and genomics will be essential for implementation.

### Maintenance of genetic variance and prediction accuracy

Long-term recurrent selection may reduce genetic variance to the point where genetic gains are limited (Jannink 2010). Indeed, genetic variance showed substantial decline over breeding cycles. The greatest decline was observed in the smallest breeding population given oligogenic architecture and high heritability. These results corroborate previous findings that showed decline of genetic variance during long-term selection is pronounced with smaller population sizes (Heffner *et al.* 2010; Müller *et al.* 2017). GARS resulted in greater loss of genetic diversity per unit time compared to PRS. This observation may account for plateauing genetic gain in GARS compared to PRS in final years. The greater loss of genetic variance in GARS *vs.* PRS can be attributed to greater selection intensity in GARS due to more selection cycles per year (Lorenz *et al.* 2011; De Beukelaer *et al.* 2017; Neyhart *et al.* 2017). Greater loss of genetic variance per unit time in GS compared to phenotypic selection has been attributed to selection and drift of alleles that are not tagged by markers in the training population, which cannot be targeted by GARS (Rutkoski *et al.* 2015). The accelerated loss of genetic variance in rapid cycle GARS highlights the need for long-term selection strategies that balance genetic gain with maintenance of diversity (De Beukelaer *et al.* 2017).

Maintenance of prediction accuracy across selection cycles is critical for long-term genetic gain (Müller *et al.* 2017). Prediction accuracies for GARS were initially high, likely due to the within-family prediction scheme, high genetic variance and similarity between the selection population and training population (Lorenz and Nice 2017). Average prediction accuracy after the first year of GARS showed substantial decline. This finding may be explained by the breakdown of LD between markers and QTL due to recombination (Jannink 2010; Müller *et al.* 2017). Phenotypic accuracy showed substantial decline only for the smallest population size, notably for the oligogenic architecture. For the polygenic architecture with low heritability, phenotypic accuracy was less affected by selection and remained proportional to the square root of narrow sense heritability (Muranty *et al.* 2015).To maintain GARS prediction accuracy over cycles, we considered a strategy in which the training population was updated and reevaluated to generate new prediction models. We found that updating the training population with the highest performing lines resulted in substantially higher genetic gain and prediction accuracy. Updating the training population has been previously shown to increase short-term prediction accuracy and genetic gain (Neyhart *et al.*, 2017). Our study indicated that updating the training population also increased long-term genetic gain. This observation can be explained by more pronounced decay in LD among QTL in long-term selection and increased genetic distance between the training population and the selection candidates, which is addressed by model updating (Jannink 2010; Lorenz *et al.* 2011).

### Next steps for implementation and optimization of GARS

Currently, the Chibas breeding program is developing multipurpose midheight (∼2 m) sorghum varieties that are simultaneously selected for a high grain yield (white hard to semihard grains primarily for human consumption) and forage quality after grain maturity. Our simulations provide guidance on several key decisions regarding resource allocation and capacity building to maximize genetic gain: (1) achieving three cycles of GARS per year (two off-season nurseries per year) will be critical to provide a higher rate of genetic gain; (2) rapid cycling GARS can provide increased genetic gain even at a modest population size (though a strategy to maintain genetic diversity will be needed if population sizes are small); (3) GARS can be cost-effective for developing countries breeding programs even at modest population size if genotyping cost under US $15; or at higher genotyping costs if larger population can be evaluated; and (4) updating the training population and prediction models using lines that capture new LD will be valuable to maintain high prediction accuracy and genetic gain. It should be noted that the simulation carried out in present study assumes that all selected parents are fertile. In an actual breeding program that employ male sterility system, however, the population segregates for male sterility. Therefore, the crossing pattern of randomly intercrossing the selected parents may differ from the breeding population in which male sterility system segregates in the population.

Future studies will need to address other aspects of genetic architecture and resource allocation that could affect implementation of GARS in small young breeding programs in developing countries. These breeding programs, which often evaluate and cross diverse germplasm, may encounter more epistasis than established breeding programs that have fixed favorable genetic interactions (Wright 1932; Cooper *et al.* 2009). In addition, changes in environment (new pests, new diseases, climate change, etc.) may shift the adaptive landscape for the breeding program and force them to restart their adaptive walk. Genome-wide prediction models with more flexible parameterization (BayesA, BayesB, random forest, etc.) (Heslot *et al.* 2012) may capture genetic architecture of small young breeding programs better than the RR-BLUP method used in this study. Another challenge is to achieve dualpurpose crop improvements targets when favorable traits are negatively-correlated due to trade-offs from source-sink relationships (Tovignan *et al.* 2016). Since the use of GS across heterogeneous target environments is poorly understood, further studies are also required to account for the effect of GxE (Heslot *et al.* 2014; Lopez-Cruz *et al.* 2015).

GS can also be applied to prioritize line advancement (*e.g.*, S0→S5) for varietal development. Genomic assisted selection on S1 and subsequent families would reduce the number of inbred families that needs to be phenotyped in replicated plots at multiple locations. It could also lower the cost of inbred line development and allow prediction for a greater number of environments and traits. While the results of the current simulation experiment can be applicable to the varietal development cycle of the recurrent selection in terms of the population size and associated costs, additional analysis may be required to test if complete phenotypic evaluation across multiple location should be done at the S2 and subsequent generations. Rigorous theoretical and empirical evaluation of genomic breeding approaches will be critical for translating technology investments into genetic gains for smallholder farmers.
